# Discourse on measurement

**DOI:** 10.1073/pnas.2401229121

**Published:** 2025-01-27

**Authors:** Arthur Paul Pedersen, David Kellen, Conor Mayo-Wilson, Clintin P. Davis-Stober, John C. Dunn, M. Ali Khan, Maxwell B. Stinchcombe, Michael L. Kalish, Katya Tentori, Julia Haaf

**Affiliations:** ^a^Department of Computer Science, Remote Sensing Earth Systems Institute, The City University of New York, New York, NY 10031; ^b^Department of Psychology, Syracuse University, Syracuse, NY 13244; ^c^Department of Philosophy, University of Washington, Seattle, WA 98195; ^d^Department of Psychological Sciences, University of Missouri, Columbia, MO 65211; ^e^School of Psychological Science, The University of Western Australia, Crawley, WA 6009, Australia; ^f^Department of Economics, The Johns Hopkins University, Baltimore, MD 21210; ^g^Department of Economics, University of Texas at Austin, Austin, TX 78712; ^h^Center for Mind/Brain Sciences, University of Trento, Rovereto, TN 38068, Italy; ^i^Department of Psychology, University of Potsdam, Potsdam 14476, Germany

**Keywords:** measurement literacy, error, policy-making, scientific reasoning

## Abstract

Measurement literacy is required for strong scientific reasoning, effective experimental design, conceptual and empirical validation of measurement quantities, and the intelligible interpretation of error in theory construction. This discourse examines how issues in measurement are posed and resolved and addresses potential misunderstandings. Examples drawn from across the sciences are used to show that measurement literacy promotes the goals of scientific discourse and provides the necessary foundation for carving out perspectives and carrying out interventions in science.

That measurement is pervasive in science is obvious. But how it is understood and practiced in science at large is inconsistent and questionable at best. Just browse through one of science’s flagship journals. In it, you might discover statements reporting that exposure to “lead is responsible for the loss of 824,097,690 IQ points as of 2015” (1), that cash recipients in “lower-income countries gained three times more happiness than those in higher-income countries” (2), or even that humans choose “numerical answers in a systematic way as though they sense within themselves—and can communicate—a reliable numerical scale for their feelings” (3).

In these cases and in many others, sensationalist measurement talk is interwoven with otherwise good science. Consistency matters. Policymakers rely on scientific publications to shape and sharpen public policy (4), while scientists rely on publications by their peers so they themselves may ascertain and advance human knowledge. To sustain trust in scientific institutions, it is imperative for measurement talk to be carried out within the bounds of sense.

Few would disagree that good measurement and good theory are indispensable partners. What is perhaps underappreciated is that many of the challenges that scientists deal with today are the same ones that scientists dealt with centuries ago (cf. 5, 6). To many, such “traditional” concerns over measurement are outmoded at best and at worst obsolete. In practice, this sentiment forgives inattention to measurement at any depth beyond the act of performing measurement itself (7, 8). Nowadays, the study of measurement has, by all appearances, become increasingly focused on evermore abstract technical problems with but slight bearing on its perennial challenges, in theory or in practice.

The real problems, however, are still there. The purpose of this discourse is to bring out into the open the theoretical and practical problems of measurement in science, to unhide them and to expose them, and to show that ignoring them begets folly and error. To this end, this discourse will examine how problems in measurement are posed and resolved. Misconceptions about measurement will also be considered in due course, and then thrown in with the wash.

In brief, this discourse makes the case for *measurement literacy*. Measurement literacy, like statistical literacy (e.g., refs. 9 and 10), promotes effective reasoning and decision-making. By contrast, measurement *illiteracy*, in its many shades, frustrates these goals. Thus, the mere existence in print of sensationalist measurement assertions in one of science’s flagship journals is cause for reflection on the current state of measurement literacy in scientific discourse.

Measurement literacy provides the necessary foundation for forming perspectives on matters in science that matter. Take the problem of reproducibility (11): Reproducible science relies on the scaling of experimental outcomes, such as effect-size scales, to measure the success of replications. Failures to understand this scaling can dramatically impact the evidential support drawn by scientists. To take another example, consider research funding decisions (12) or even public health policy decisions (13). In both cases, measurement literacy matters for understanding and evaluating decisions that rely on aggregate performance indices and numerical-scoring mechanisms.

To understand the problem of literacy in measurement is to understand, in some degree, measurement. We therefore begin this discourse with a primer on measurement and its goals, requirements, and problems. Among the most important of its problems is the problem of its justification, which we turn to first. Measurement literacy gives the wayfaring scientist the foundation to establish trade routes through uncharted seas, connecting landmarks with new lands. It also provides the scientist with means necessary for understanding and reasoning about known and new seaways and ports, and chronicling it all in a way that can be acted upon and believed. While so too is literacy in measurement crucial to charting passage through the high seas, seafaring is a dangerous business; currents change and landmarks sink. Risk and error abound at sea.

Many important questions about measurement are omitted from consideration in this discourse. For example, problems for the development and application of measurement methods and techniques are not covered here; problems for the design and deployment of instruments and tools of metrology are also beyond the scope of this discourse. Similarly, no attempt is made to address important but technical questions about the relationship between measurement and, say, statistical inference or causal discovery algorithms.

This discourse also omits a mathematical treatment of measurement. The study of measurement is already fortunate enough to possess an impressive library of technical treatises on the subject (see, e.g., refs. 14–22). No one wants another one. While formal methods play an important part in the study of measurement, they do not define its problems.

## Measurement, In Brief

To measure something is, in one way or another, to represent it. What is unfamiliar and perhaps unwieldy is represented by what is familiar and convenient. Historically, the real number system has spoken to the desire for familiarity and convenience in carrying out the business of science. It equips scientists with a powerful medium for transforming and communicating information (cf. 23, p. 60; 15, p. 50).

Consider a set of rigid rods. Cursory inspection is sufficient to determine that some of the rods are longer than others when placed side-by-side. Associate to each rod a real number representing its length. What is obtained is a *measurement scale*.

The scale is hardly unique. A rod can be measured in inches, yards, or miles—or even, say, centimeters, meters, or kilometers. But not any assignment of numbers will do when it is length that is being measured. The scale for measuring length is but one from among a family of scales for the rigid rods related to each other by the type of requirements that length imposes on its representation.

What *is* unique—and what historically has been the subject of intense systematic study—is a measure’s *scale type*. It is the common denominator, or defining property, among all representing measurement scales. For attributing length of the rigid rods, the common denominator requires ratios between every pair of rods to be invariant across all representing scales—and so the type of scale for measuring length is called a *ratio scale*. Each scale can be obtained from any other by a positive linear transformation—and so 2.57 centimeters rings up at 1 inch, 12 inches at 1 foot, and so forth. Thus, *up to multiplication by a positive real-valued constant*, the scale for measuring the length of rigid rods is *unique*. Put concisely, the measurement scale is unique up to *choice of unit of measurement*.

When it is not length being measured, but some other attribute, the requirements that the attribute’s measurement imposes on its representation might change. Any scale obtained by measuring the attribute would therefore be subject to the requirements of a distinct scale type. Inspect the rods once more. Plain to the touch is that some rods feel warmer than others. Placing the rods in rank order of warmth forms an *ordinal scale*. Its common denominator is uniqueness up to any scale preserving the relative ordering of rods ranked by warmth.

When the attribute of interest is, say, the manufacturer date of rods, then any assignment of numbers for measuring dates of the rigid rods forms an *interval scale*. Uniqueness up to multiplication by a positive scalar and addition of a real-valued constant is the common denominator of any scale measuring the dates of the rods (fancy talk: *up to positive affine transformation*). And so on. Table 1 summarizes the traditional classification of scale types credited to Stevens (24) and subsequently developed extensively over the second half of the 20th century.

**Table 1.** Scale types, common admissible transformations, and examples

Scale	Transformation	Examples
Absolute	x↦x	Relative frequency, count
Ratio	x↦λx, λ>0, λ real number	Duration, length, mass, dosage, reaction rate, electric current
Interval	x↦λx+μ, λ>0, λ,μ real numbers	Calendar date, temperature, potential energy, cardinal utility
Ordinal	x↦ϕ(x), ϕ strictly increasing on real numbers	Letter grades, triage rank, air quality, social dominance
Nominal	x↦ϕ(x), ϕ bijection on real numbers	Treatment groups, species, genotype

The scale type of an attribute like length is determined by abstract requirements that the attribute’s measurement imposes on its representation. But what grounds can be given for ascribing a scale type to an attribute in the first place? What endows length with a ratio scale? The question over a scale type’s *justification* is a burning question for the working scientist. We enter into thorny territory. Clear thinking will clear the way.

## Measuring It

Track changes of liquid volume inside a mercury thermometer by tick markings along its side. Two entries are logged: one upward change from 30°F to 31°F and one downward change from 45°F down to 44°F. What grounds are required to claim that in the two cases the temperature has changed by the same amount? For that matter, what is the basis for the claim’s presumption that one and the same attribute is being measured in the first place? These are hard questions to answer in any completely satisfactory way. But these questions, and others like them, are among those that working scientists deal with on a routine basis, whether they come to terms with them or not.

These questions, and others like them, concern the problem of measurement “validity” (e.g., ref. 25), sometimes referred to as “nomic measurement” (5) or “coordination” (26). This is the problem of *justifying* the existence and form of a functional relationship between indices obtained by performing a procedure and the magnitudes of an attribute purportedly being measured. It is to be distinguished from purely formal problems concerned with the mathematical description of measurement scales (e.g., ref. 18).

It is obvious that trying to establish that some structured index is somehow the true measure for an attribute by direct verification is out of the question. In practice, coordination is justified through an iterative process that leverages various theoretical, practical, and empirical arguments against each other (e.g., refs. 5 and 27). The historical case of thermometry provides a crisp illustration of how such a process can unfold (5).

A presumption of quantifiability requires *reasons*. The scientist bears the burden of establishing a basis for explaining how the attribute is, or could be, related to other established attributes or measurement practices, and in some cases, of demonstrating how its application contrasts with its use in ordinary language. To return to the case of length, there are many procedures for its measurement. The relationships among these procedures are well known by scientists. Physical theory specifies how they are related to other physical quantities such as acceleration. And so on.

But sometimes the conceptual issues are not so clear. Consider “extroversion”—or “*extra*version,” according to Carl Jung—one of the *Big* five factors of personality (28). What warrants its current numerical representation on a ratio scale? Looking at the ordinary-language understanding and use of the term, comparative statements such as, “Anna is more extroverted than Debbie,” comport with its use in third-person ascriptions and first-person avowals (see refs. 29 and 30). But a proclamation that “Anna is *ten* times more extroverted than Debbie” runs afoul of ordinary usage. The guidance provided by ordinary usage can be supplemented by introducing a technical definition of extroversion that enjoys value over and above tracking expressions typically attributed to extroversion, such as, “I like to go to parties,” “I like people,” and the like. In this way, cogent grounds for treating extroversion as a ratio-scaled attribute or dimension might be established (30–33).

To illustrate the design of a technical concept, consider the measurement of competitive ability among organisms—fitness. Nebulous and tautological conceptions of fitness (see ref. 34, chapter 2) can be sharpened into a ratio-scaled representation of relative population frequencies. This representation turns out to facilitate the derivation of well-known selection equations (35) and the formulation of precise definitions of phenomena such as gene–gene interactions (for a detailed discussion, see ref. 36).

Conceptual considerations bear on empirical merits of theories and models that postulate the measured attribute. The study of measurement has led to the identification of nontrivial constraints that can be used to put to the test the claim that a given attribute is amenable to a ratio-scale representation (e.g., refs. 37–43). Some of these tests will be considered later on in the *Error* section. A well-known example from psychology is signal detection theory (SDT, 44). Its ratio-scaled attributes of *discriminability* and *response bias* have been validated by its ability to successfully describe and predict people’s judgments in a wide variety of domains (e.g., recognition memory; see refs. 45 and 46).

Another well-known example, this time coming from the intersection of psychology and economics, is helpful to understand the difference between conceptual and empirical considerations. Prospect Theory (47) postulates a “loss aversion” attribute defined in terms of people’s appreciation for lotteries including both gain and loss outcomes (48, 49). The sharpness of this definition notwithstanding, Prospect Theory is often found to underperform relative to rival theories that do not include loss aversion as an attribute (e.g., ref. 50).

Predictive success is generally acknowledged to be but one of many factors that can figure into a theory’s support. This includes establishing the validity of measurements. Quantities that predict might not be measures of anything. To see this, consider a survey consisting of one question, “How many records by the Beach Boys have you purchased?” No doubt a pronounced correlation would be established between the response variable for the survey question and other quantities for attributes of survey subjects, like age or weight, and so could be used to predict negative health outcomes like arthritis, heart disease, or senility. Yet, it stands to reason that there is no such thing as the Beach-Boy-Health Index, however it is you stipulate the form of the correlation.

Next, turn to the assertion that ratings of life satisfaction on a 10-point scale are predictive of significant life events, such as quitting a job, ending a romantic relationship, and so on. It is on this basis that (3) make the striking claim quoted at the outset of this article, namely, that humans “sense within themselves—and can communicate—a reliable numerical scale for their feelings.” This conclusion is unwarranted.

Also unwarranted is the presumption that a reliable correlation between two indices provides evidence that they measure the same thing. Consider, for example, using a balance to measure the mass of several stacks of identical cubical blocks. You will notice that the results are linearly related to the heights of those stacks as measured by a ruler. Yet rulers and balances measure different things. The same obviously applies to the indices coming from Beach-Boy-fanhood and health surveys. Guttman summarized it best when stating that “*Correlation does not imply content*” (51). Being able to avoid this kind of unwarranted conclusions is crucial in the current era of Big Data, as the vast majority of correlations found in large datasets are spurious (52).

That said, Guttman’s aphorism does not extend to “negative” claims that distinct indices measure different things. In fact, there is a long-standing practice in using contrastive measures (e.g., single and double dissociations in factorial experimental designs) in the localization of mental functions in the brain (53). But obtaining diagnostic results through this way can be easier said than done, as some conclusions can only be drawn when representing attributes in certain ways (e.g., refs. 54–56). This ambiguity will be discussed in greater detail in the upcoming *Meaningfulness* section.

At least in the social and behavioral sciences, the lineage of some of the unwarranted claims discussed so far can be traced back to the objective of *construct validity* as popularized by Cronbach and Meehl in the 1950s (for reviews, see refs. 25 and 57). Constructs are at once conceived to be abstractions (that “describe,” “summarize,” “encapsulate”) and themselves objects for scientific inquiry (to be “detected,” “explored,” or “manipulated;” see refs. 58, 59, 60).

Construct validity is attractive to because it speaks to scientists’ general desire for measures to be as *theory-agnostic* as possible. But the extent to which measurements are theory-laden is sometimes a point of contention (e.g., the famous Koopmans-Vining debate of the 1940s; see ref. 61). Regardless, there is a real risk for this desire to devolve into a poor understanding of the measures being used, which can lead both scientists and policymakers astray and likely lead to social harm.

Recent developments in the study of eyewitness identification help to illustrate the stakes. For several decades now, researchers and policy-makers have been preoccupied with the effectiveness of lineup procedures performed by police departments and their contribution to the risk of wrongful conviction. Numerous studies comparing different lineup procedures, using measurements of so-called “probative value,” drew the conclusion that lineups formed sequentially are superior to lineups in which individuals are presented side-by-side (62). In time, these findings shaped the guidelines issued by the U.S. Department of Justice (see ref. 63, p. 594), and by 2013, one-third of U.S. police precincts used sequential lineups (64). Later work revealed the theoretical and empirical shortcomings of measurements of “probative value” (e.g., refs. 64 and 65). Crucially, subsequent research relying on SDT-based measures overturned the claimed superiority of sequential lineups (64).

A firm grasp of what goes into justifying measurement claims also helps scientists to have a clear reading on what can be properly justified. In the same way that the evidence obtained can only go so far in pinning down the details of existing theories, there are limitations when it comes to establishing a scale type. How to navigate these challenges will be the focus of the next section.

## Meaningfulness

Measurement literacy helps scientists in detecting misleading, imprecise, or unsubstantiated quantitative claims. It provides standards for scientific discourse and in particular for *meaningful* communication—a technical qualification to be given sense in what follows.

Consider the striking statement about happiness from the outset of this discourse, according to which residents of lower-income countries who are given USD $10,000 gained “three times more happiness than those in higher-income countries” (2). That statement is striking, not because it implies happiness is amenable to scientific examination, nor because it implies that happiness can be measured and quantified. It is striking because it presumes that happiness admits quantification on a ratio scale. That would mean that happiness is a quantity like length.

While various types of evidence might be proffered to ground the claim that happiness conforms to a ratio scale, no such evidence exists as of the writing of this discourse. There is no evidence that the many measures of happiness tabulated by Veenhoven (66) are related by positive affine transformations. Even analytic arguments are wanting; it is hardly self-evident that happiness is a singular attribute admitting representation on a ratio scale, and one that can be compared between two groups of individuals or even by one individual at different moments of life (cf. 67). These challenges are not unique to the study of happiness and confront attempts to quantify well-being at individual and societal levels more widely (e.g., ref. 68).

If happiness fails to be ratio-scaled, then the striking statement’s publication has the potential to mislead even those with the best intentions. Consider a scientist acting as advisor for a policy-maker. Suppose the policy-maker wants to redistribute economic aid between two countries. Based upon the reporting in ref. 2, the scientist would reasonably assume any reduction in happiness in the former country would be outweighed by a corresponding three-fold increase in happiness in the latter.

Yet, should happiness fail to be quantifiable on a ratio scale, follow-up studies that quantify happiness by “rescaling” the unit of measure might very well find that the implementation of the policy-maker’s program to be a foreign policy failure of the third degree, not only because it did not increase happiness three-fold in the target country but also because it drove down overall happiness in the target region. The policy-maker’s predicament is not out of the ordinary; consider, for example, policy planning and evaluation in environmental conservation (69, 70) or border security (71).

The statement runs afoul of a standard that scientific publications are ordinarily expected to observe as harbingers of knowledge. More specifically, the statement about happiness from the outset of this discourse falls short of the technical criterion of *meaningfulness*, because, in fact, its truth value can vary with the scale that is chosen for measuring happiness. In rough yet somewhat abstract terms, a statement is said to be *meaningful* just in case its truth or falsity is independent of what scale is chosen to measure the target attribute from among those related by scale type. For example, the assertion that diamond is one-hundred times harder than gold is not meaningful. Although the assertion may be true given one scale for hardness (e.g., Knoop’s), it is false on others (e.g., Moh’s). Similarly, the statement that a patient’s temperature at noon is 2% higher than it was at noon yesterday is not meaningful.

Scientists and policymakers alike trust that scientific publications implement reliable checks and balances protecting against promulgation of shaky science. A published hypothesis that fails to be meaningful can fail to replicate if the measurement scale that is used is different from the one used for the publication—and so threatens to mislead scientists and policymakers and thereby needlessly expose the public and even the international order at large to harm and discord. Yet a published hypothesis that successfully replicates can fail to be meaningful, a situation that, if handled inappropriately, can propagate and perpetuate misconceptions. Measurement literacy is requisite for scientists to be responsible and trusted ambassadors of knowledge.

The difference between statements that are meaningful and those that are not can be subtle. One subtlety concerns the “problem of coordination” discussed earlier in the *Measuring It* section: the unknown functional relationship between an index and the attribute that it is purported to measure. Because of this gap, a statement about the index can be meaningful at the same time that a counterpart statement about the attribute is not.

**Fig. 1.**
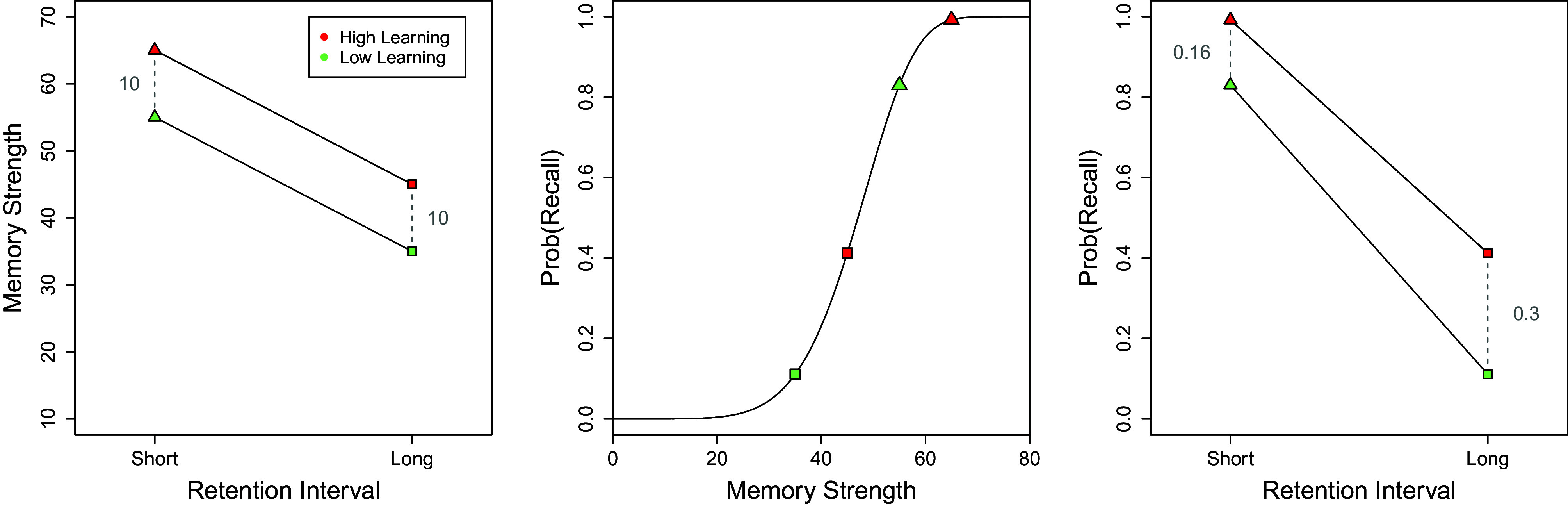
*Left* panel, Illustration of how additive (ANOVA) effects at the level of a quantitative attribute (memory strength; see *Left* panel), by virtue of a nonlinear relationship (center panel), implies an interaction effect at the level of an outcome variable (probability of recall; see *Right* panel).

Consider a memory experiment where participants study a list of words under two learning regimens—call them “high learning” and “low learning.” Later, after different retention intervals—“short” for some and “long” for others—they are asked to recall what they have learned. The average recall rates obtained with this 2 × 2 factorial design, illustrated in the *Right* panel of Fig. 1, show an *interaction effect* in the ANOVA sense. More specifically, the difference between effects of short and long retention intervals on recall is smaller in the high-learning condition (0.16 difference) than it is in the low-learning condition (0.30 difference). This difference measure lies on an absolute scale, and so, is *unique* in the strongest sense that it is the only scale belonging to its scale type (Table 1). Therefore, there is no problem with statements like, “*Recall rates* decreased faster in the low-learning condition than they did in the high-learning condition.”

Broadly speaking, recall rates are of interest to cognitive scientists because they help to illuminate the cognitive processes associated with mnemonic faculties (e.g., refs. 72 and 73). But while scientists are generally willing to postulate “memory strength” attributes belonging to an interval scale type (e.g., refs. 45, 74, and 75), presuming anything more than an ordinal scale about their relationship with something like recall rates would be contentious at best (76; but see also refs. 77 and 78).

Hence, in the present case, to say that some memory-strength attribute decreased faster in the low-learning condition than it did in the high-learning condition would fail to be, by definition, meaningful. Fig. 1 illustrates why. The center panel shows that outcome of nonlinear monotonic transformation of memory strengths into recall probabilities. In turn, the *Left* panel illustrates the average memory strengths obtained across the different experimental-manipulation conditions. The effects of these experimental manipulations are additive (i.e., there is no interaction). But when transformed into recall probabilities, these effects are no longer additive (i.e., there is an interaction). Recent literature reviews in psychology show that researchers are generally unaware of these subtleties (79) and are often found drawing conclusions that fail to be meaningful (80). In part, there seems to be a confusion between the replicability of an outcome, such as the interaction effect in Fig. 1, and the meaningfulness of the measurement statements surrounding it. In truth, successfully replicating an effect has no bearing on the meaningfulness of a measurement statement (see refs. 64 and 81).

Inattention to measurement basics can compromise effective use of aggregate indices in scientific discourse and policy-making. Statements that compare arithmetic means of measures for an attribute on an ordinal scale might fail to be meaningful if the rank ordering given by the arithmetic means fails to be preserved by some nonlinear strictly increasing transformation of the scale. Thus, ranking research grant applications by their averages might depend on the choice of ordinal scale used for rating them. By contrast, using *geometric* means or medians would be independent of the choice of ordinal rating scale, as ranking by means would be preserved by any nonlinear strictly increasing transformation of rating scale (82, 83). In this context, measurement literacy affords critical insight into the conditions and uses for combining measures to direct intelligent reasoning and decision-making (see ref. 84, for a detailed discussion). Measurement literacy likewise provides guidance for using parametric and nonparametric methods in hypothesis testing (83–85).

While we have been content in this discourse with an informal treatment of the criterion of meaningfulness in measurement, there is an extensive literature giving rigorous treatment to its precise formulation (13, 15, 16, 20, 86–93). Sometimes treatments of meaningfulness use the term “meaningless” for statements that fail to be meaningful in the technical measurement-theoretic sense. We have refrained from this terminology in view of the historical use of the word and its variants by logical positivists as a slur.

A more controversial aspect of meaningfulness concerns its relationship with statistical inference. When proposing his famous classification of scale types shown in Table 1, Stevens argued that the scale of one’s data determined which statistical methods are “permissible.” According to Stevens’ doctrine, the analysis of nominal and ordinal data requires nonparametric methods (provided that they are available, which is not guaranteed for experimental designs of even modest complexity; e.g., see ref. 55), whereas the analysis of interval and ratio-scaled data permits use of parametric methods (21, 94–96). Since its inception, Stevens’ doctrine has been subject to intense debate. Its critics live by Lord’s Word, “the numbers don’t know where they came from” and therefore deny that measurement scale types place any limitations on statistical inference whatsoever. To put it differently, whether or not certain numerical assignments are measures of anything has no bearing on their legitimacy as data (97–101).

The rigidity of this doctrine is hard to miss. Take intelligence measurement. Consider the hypothesis that the distributions of intelligence of two groups differ. Assume that intelligence admits quantification by scores on an ordinal scale and that the measurement scale is valid for the different groups of individuals that it is applied to. Under these assumptions, nonparametric tests can be used to evaluate the differences between the distributions, on the basis of which the conclusion may be validly drawn that the distributions do or do not differ, as the case may be. But, in general, it is invalid to conclude that, on average, one group is more or less intelligent than another group on the basis of differences in the arithmetic means. For such a conclusion to be meaningful, the scale must have properties stronger than being ordinal (see ref. 102).

## Design

One subtlety in the memory example discussed earlier (Fig. 1) is that effects like interactions do not stand on their own. Rather, their standing depends on all the other effects that, together, characterize a data pattern (e.g., ref. 103).

In the case of ANOVA, it turns out that the claim that the smallest of its effects (main effects, interactions) exists (i.e., that it is nonzero) does not hold true across all possible ordinal scales. In other words, it fails to be meaningful (see ref. 104). The immediate implication from this insight is that the meaningfulness of an ANOVA effect can be guaranteed by making sure that it is not the smallest one. This can be achieved by fashioning the design of the experiment accordingly. For instance, one could select the experimental factors, say the learning regimen, so that one of the main effects is the smallest.

This example illustrates one of the many ways in which measurement literacy can contribute to the development of successful study design and successful research programs more generally. Yet it is our contention that scientists generally underestimate the importance of deliberating about measurement in study design.

Take the case of random sampling (whether simple or stratified). Textbook introductions discuss results showing that random sampling guarantees unbiased estimators for many quantities of interest, such as population means, population totals/sums, and differences between group means (105, 106). But these results, as useful as they might be, are limited in scope. They apply only to *quantities*—and of a specific type, namely, *means* and *sums*, rather than medians, minima, or maxima, say. If the measurement scale type of the attribute of interest is at best ordinal, then sums and averages are unlikely to be quantities of direct interest. But changing estimates of interest is not as simple as it looks, for the simple reason is *no sampling methodology* that can guarantee their unbiased estimation (107).

Just as random sampling guarantees that there is an unbiased estimator of the population mean, so too do randomized experiments guarantee that there are unbiased estimators of average-treatment effects (108). But again, the focus on quantities and on *mean* treatment effects, in particular, is critical. If the outcome of interest is not a quantity at all, or if it is a merely ordinal or nominal variable, then a researcher might be interested in some other form of a causal effect that is not best estimated via a randomized experiment. This lesson is important because there is a growing trend to prioritize randomized experiments over observational studies in the social and biomedical sciences (108).

For examples of how measurement-scale considerations can inform study design, look no further than to applications of model-based sampling. This consists of using domain-specific knowledge to build a statistical model of the population and then choose a sampling scheme that permits the estimation of the parameters of that model. This approach has been successfully employed in agriculture, medicine, and ecology, among other fields (e.g., see refs. 109 and 110).

Imagine someone trying to plan a new study on the impact of exposure to lead on IQ (1). A model-based approach might use past data suggesting that the relationship between blood-lead levels and IQ is roughly log–linear (111). The regression coefficient in that model could then be used to estimate total IQ loss. If the goal is to estimate that coefficient (and hence, total IQ loss) precisely, one should systematically sample Americans with the highest blood-lead levels, not sample randomly (see ref. 112).

This sampling solution would need to be reconsidered though, if the goal is to rely on IQ to make claims about intelligence. Given that IQ is at best an ordinal index of intelligence (113), hypotheses about population medians or minima might be of greater interest to researchers than hypotheses about population means or sums, as the former involve meaningful measurement claims whereas for the latter, only in specific instances. In this context, scientists would benefit from considering statistical models (e.g., ordinal regression) that will help them make meaningful estimates with respect to the appropriate measurement scale type.

## Error

Measurement literacy is crucial for navigating measurement errors intelligibly, and in turn, leveraging them effectively in testing and developing scientific theories.

Talk of error is present in pretty much every area of science. But error talk does not speak for itself—it requires some standard to be in place. The proverbial table leg proclaimed to be off by one inch is presumed to have some definite length by which the measure errs. But what of the case where the length of a table leg is measured multiple times throughout the day? To attribute error to each recorded measurement is to presume *one length* of the leg. Nothing stands in the way of interpreting any and all discrepancies to be true expressions of the attribute’s “natural variation”—that is, to impute many lengths to the leg. True, nothing does; but pursuing this would not be helpful (114).

The table leg example illustrates an important insight, namely, that in any context in which measurement is said to take place, there are accepted background assumptions that set general rules on how to attribute error (115). To be clear, errors of measurement are *not* exceptions to the general rules set; they are stipulations about the veracity or fidelity of observational reports. Theories include assumptions (e.g., Newton’s third law of motion, the law of linear thermal expansion) which stipulate that any observational report at odds with them is in error. To fix ideas, consider a case where leg *a* is reported to be longer than leg *b*, followed by another report that leg *b* is longer than leg *a*. These two reports are only at odds with each other, with at least one of them in error, if one assumes that (i) length is invariant over time and (ii) that the relationship expressed by “longer than” is an asymmetric relation. For more vivid examples of error adjudication, consider the field of paleontology, where models are routinely relied on to correct or debias fossil records (116).

Distinct theories rest on different assumptions and therefore might very well disagree over what counts as an error. Consider how classic and quantum theories differ over the way in which attributes are treated and their relationship with measurement procedures. Classical physics frames measurement as a process in which the true magnitude of an attribute becomes known. In turn, quantum theory states that there is no true measure by which we err—magnitudes are “created” by the taking of measurements (117). Outcomes incompatible with one theory’s assumptions are acceptable according to the other. In other words, the appeals to error made by the two theories are very different.

Measurement errors are obtained when engaging in a process of reconciliation between the observations and the assumptions being upheld (118). Return to the table leg example. Assuming that its length is the same at all times that measurements were taken, then errors can be estimated by adjusting each recorded value so that they perfectly agree on some quantity *L*. Here, *L* is no longer being treated as directly observable, but as a nonobservable quantity whose estimation is a function of the reconciliation process (e.g., minimization of squared errors; see refs. 114, 118, and 119).

The quantification of error provided by such a reconciliation provides important grist for the intellectual mill. When comparing distributions of errors, some might be perceived as negligible or tolerable, whereas others might be too large or systematic to be left wanting for an explanation. Take the case of Laplace’s study of the solar system, in particular the motions of Jupiter and Saturn. When observing irregularities, Laplace weighed the merits of attributing them to error vis-à-vis unaccounted causes. When errors were deemed too large, he would pursue the latter account. In the cases of Jupiter and Saturn, Laplace was able to explain the observed irregularities in their motion by appealing to the mutual gravitational attraction of the two planets (120).

This appraisal of theories and hypotheses through the quantification of error brings us to a point touched upon earlier in the *Measuring It* section, namely the possibility of conducting tests that speak to the basal hypothesis that a given attribute is amenable to a ratio-scale representation. For the longest time, the deployment of these tests was frustrated by a deficit of work integrating errors into statistical-inferential machinery (be it frequentist, Bayesian, or whatever). But without the possibility of error, a single recalcitrant observation is enough to undermine the presumed measurability of an attribute. Fortunately, recent developments have resolved many of these challenges, creating new opportunities for theory testing and development (e.g., refs. 41, and 121, 122, 123).

The success of theories can be determined by testing the constraints imposed by the attributes that it postulates. Take the notion of strength of preference or utility that underlies a large family of theories, including notable members such as Prospect Theory (47). According to this family of theories, people’s preferences conform to a number of constraints, the requirement of transitivity being one of them (124–126). Under the appropriate experimental designs, the different constraints that preferential choices must satisfy become very strict, introducing the possibility of strong-inference testing (127). To reject these constraints is to reject a large family of theories altogether (124–126).

One attractive feature of these tests is that they offer the possibility to cast routine hypotheses under weaker scaling assumptions. For instance, instead of assuming a *linear* relationship between experimental factors and the data, as done when using off-the-shelf methods such as ANOVA, one can merely assume that there is a monotonic relationship (see ref. 55). This reliance on weaker assumptions such as monotonicity directly addresses concerns with the meaningfulness of effects, as illustrated earlier in our memory example (Fig. 1).

In some cases, due care in the handling of errors includes offering a principled distinction between measurement error and the natural variability or stochasticity of attributes (128, 129). As an illustration, consider a scenario where a person expresses a preference for *a* over *b* at a given point, but later claims to prefer *b* over *a*. These discrepant observations can be plausibly said to reflect a change in that person’s (true) preferences (e.g., refs. 124 and 130). Now, contrast this scenario with the earlier table leg example. Because length is presumed to be an invariant attribute in most applications, one should expect an analogous set of observations to be attributed to the presence of error (e.g., ref. 119).

Failing to acknowledge the errors in our ways can lead to mischaracterizations (e.g., refs. 130 and 131). But scientists can also fail to keep error in its place. Take the widespread practice in the social, behavioral, and health sciences of gathering so-called measurements of human feelings. Despite (3)’s claims that people somehow appear to reliably operationalize their feelings over numerical scales, there is no sense in which these measurements can be in error. Can someone be said to be *mistaken* about how sad they are, or about how much their headaches? Leaving aside cases of self-deception, and regardless of the fickleness of these feelings and sensations, the answer is arguably in the negative (for discussions, see ref. 132, Chapters 5 and 8).

## Conclusion

Measurement literacy is crucial for effectively navigating and advancing scientific discourse. A working understanding of its problems, requirements, and goals affords the working scientist with the foundation necessary for thinking things through, from problems in validity, inference, experimental design, and error to policy-making and communication.

In recent years, discourse in science, especially in the social and behavioral sciences, has weathered numerous crises, from the reproducibility crisis (e.g., ref. 133) to the theory crisis (e.g., ref. 134), as well as myriad attempts to address them, from mandating preregistration to calls for more theory-driven hypothesizing. To address such crises and evaluate proposals to address them, it is necessary for measures to be taken to reinvigorate measurement literacy in discourse on science.

This discourse is a call to action.

## Data Availability

There are no data underlying this work.
